# Exploring the Mechanism of H_2_S Synthesis in Male Bactrian Camel Poll Glands Based on Data Independent Acquisition Proteomics and Non-Targeted Metabolomics

**DOI:** 10.3390/ijms25147700

**Published:** 2024-07-13

**Authors:** Bao Yuan, Quanwei Zhang, Bohao Zhang, Jianfu Li, Wenli Chen, Yu Zhao, Weitao Dong, Yong Zhang, Xingxu Zhao, Yuan Gao

**Affiliations:** 1 College of Life Science and Technology, Gansu Agricultural University, Lanzhou 730070, China; 17693462916@163.com (B.Y.); li18419220018@163.com (J.L.); c2593840952@163.com (W.C.); zhaoyudc@163.com (Y.Z.); zhangyong@gsau.edu.cn (Y.Z.); zhaoxx@gsau.edu.cn (X.Z.); 2 Gansu Key Laboratory of Animal Reproductive Physiology and Reproductive Regulation, Lanzhou 730070, China; zhangbhgs@163.com (B.Z.); d.wt2008@163.com (W.D.); 3 College of Veterinary Medicine, Gansu Agricultural University, Lanzhou 730070, China

**Keywords:** poll glands, DIA proteomics, non-targeted metabolomics, hydrogen sulfide

## Abstract

During estrus, the poll glands of male Bactrian Camels (*Camelus Bactrianus*) become slightly raised, exuding a large amount of pale yellow watery secretion with a characteristic odor that may contain hydrogen sulfide (H_2_S). However, whether H_2_S can be synthesized in the poll glands of male Bactrian Camels and its role in inducing camel estrus remains unclear. This study aimed to identify differentially expressed proteins (DEPs) and signaling pathways in the poll gland tissues of male Bactrian Camels using data independent acquisition (DIA) proteomics. Additionally, gas chromatography–mass spectrometry (GC-MS) was performed to identify differentially expressed metabolites (DEMs) in the neck hair containing secretions during estrus in male Bactrian Camels, to explore the specific expression patterns and mechanisms in the poll glands of camels during estrus. The results showed that cystathionine-γ-lyase (CTH) and cystathionine-β-synthase (CBS), which are closely related to H_2_S synthesis in camel poll glands during estrus, were mainly enriched in glycine, serine, and threonine metabolism, amino acid biosynthesis, and metabolic pathways. In addition, both enzymes were widely distributed and highly expressed in the acinar cells of poll gland tissues in camels during estrus. Meanwhile, the neck hair secretion contains high levels of amino acids, especially glycine, serine, threonine, and cystathionine, which are precursors for H_2_S biosynthesis. These results demonstrate that the poll glands of male Bactrian Camels can synthesize and secrete H_2_S during estrus. This study provides a basis for exploring the function and mechanism of H_2_S in the estrus of Bactrian Camels.

## 1. Introduction

The Bactrian Camel (*Camelus Bactrianus*) is a unique and advantageous livestock resource in desert and semi-desert regions, with unique adaptability to adverse environments such as cold, drought, and heat, and has important economic and research value [1]. However, in recent years, the population of Bactrian Camels has continued to decline, significantly restricting the development of the camel industry and local economies. This decline is primarily due to an incomplete understanding of the breeding mechanism of Bactrian Camels [2]. The poll glands of Bactrian Camels, also known as the “occipital glands” or “neck occipital glands”, have a structure similar to that of male Dromedary Camels. The glandular parenchyma is inverted in a “V” shape, located in the dermis on both sides of the first cervical vertebra, and is composed of multiple almond-colored pyramidal lobules [3,4]. During the rutting season, male Bactrian Camels exhibit external sexual characteristics such as foaming at the mouth, making a “beep” sound, grinding teeth, running, competing for females, itching, and loss of appetite. The poll glands of male Bactrian Camels secrete a light brown or amber viscous fluid with a foul odor, referred to as “poll gland secretion” or “occipital gland secretion”. The more intense the male camel’s estrus, the larger the poll glands develop, and the more mucus they secrete. Enhanced functioning of the poll glands and their increased secretion in male camels are key factors affecting the reproduction of Bactrian Camels. Studies have shown that the development of the poll glands and their increased secretion can enhance male camels’ sexual activity, stimulate female camels to estrus, and induce female camels to mate [5]. However, the mechanism by which the poll glands of male camels affect reproductive activity is not fully understood at present.

Hydrogen sulfide (H_2_S), a gaseous signaling molecule, is a colorless gas with a foul odor [6]. Research has shown that the biosynthesis of H_2_S in mammals uses L-cysteine (L-Cys) as a substrate and is catalyzed by four enzymes, namely cystathionine-γ-lyase (CTH or CSE), cystathionine-β-synthesis (CBS or CBSL), 3-mercaptopyruvate sulfurtransferase (3-MST), and cysteine aminotransferase (CAT) [7]. These H_2_S synthesizing enzymes exhibit specific distribution in organisms, with CTH and CBS primarily found in the cytoplasm, while 3-MST is expressed in both the mitochondria and cytoplasm [8]. The dissolved H_2_S is weakly acidic and exists in the equilibrium state of H_2_S ↔ HS^−^ ↔ S^2−^. H_2_S has a half-life in the body ranging from a few seconds to a few minutes [9]. At physiological pH, H_2_S and HS^−^ are in equilibrium at a ratio of 28% to 72% [10]. As the third gas signaling molecule after nitric oxide (NO) and carbon monoxide (CO), H_2_S participates in regulating various biological processes, including vasodilation, smooth muscle relaxation, and neuronal protection [11,12]. Endogenous H_2_S can participate in the body’s metabolism through various pathways. Due to its ease of diffusion, H_2_S can be oxidized in mitochondria and ultimately converted into sulfites and sulfates, which are excreted from the body as free or conjugated sulfates in urine [13,14]. Although the importance of H_2_S in brain, vascular, and cardiac functions is known, its transport mechanism has not been well explained. Some studies suggest that H_2_S is a highly lipophilic molecule that can freely penetrate all types of cells, while others indicate that aquaporins such as aquaporin 1 (AQP1) and aquaporin 4 (AQP4) can serve as H_2_S transporters [15,16]. However, the study of H_2_S in the poll glands of Bactrian Camels remains unclear. This study hypothesizes that the key enzymes regulating H_2_S synthesis are expressed in the poll gland tissues of camels. Elucidating the molecular mechanism of H_2_S synthesis in the poll glands would provide a theoretical basis for the reproductive regulation of Bactrian Camels.

This study utilized data independent acquisition (DIA) proteomics and non-targeted metabolomics analysis to systematically identify the key enzymes regulating H_2_S synthesis in the poll gland tissues of male Bactrian Camels and revealed that H_2_S plays an important role in regulating seasonal estrus in male camels. This study provides a basis for further exploration of the physiological functions and regulatory mechanisms of H_2_S in the estrus and reproduction of Bactrian Camels.

## 2. Results

### 2.1. Identification and Annotation of DEPs Based on DIA Proteomic Data

**Figure 1:**
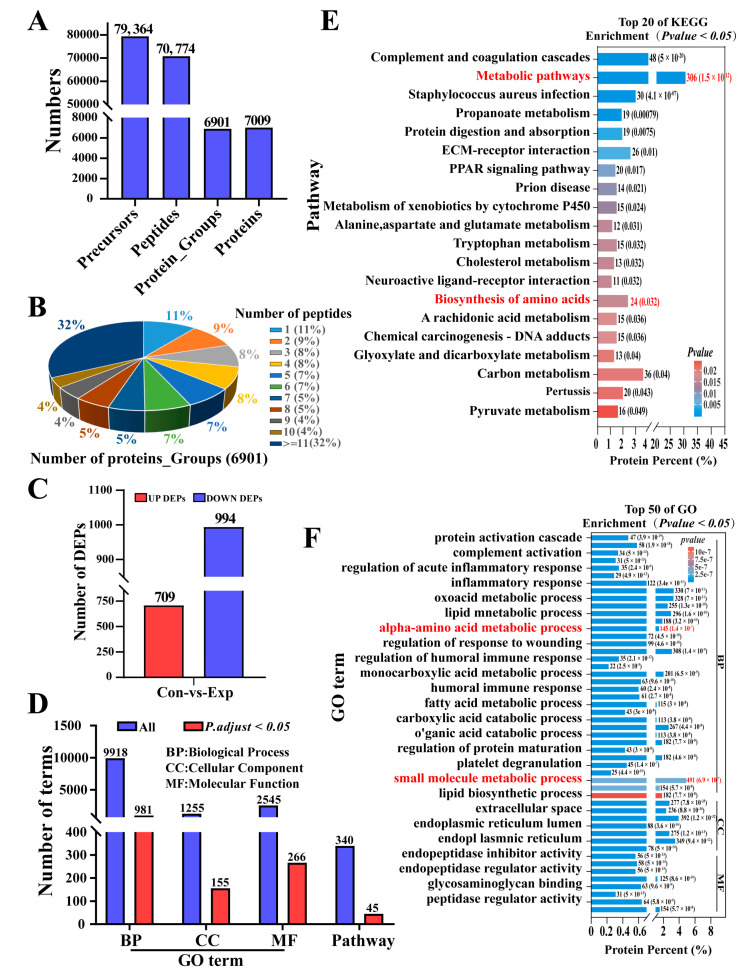
Identification and annotation of DEPs based on DIA proteomic data in poll glands during estrus. (**A**) Number of recognized precursors, peptides, and proteins. (**B**) Peptide number distribution and proportion of proteins. (**C**) The number of significantly upregulated and downregulated proteins in poll glands during vigorous estrus. (**D**) GO annotation and pathways of the 1703 DEPs. BP: biological process. MF: molecular function. CC: cellular component. (**E**) The top 50 enriched biological processes in GO annotations of the DEPs. (**F**) Top 20 enriched signaling pathways of the DEPs. Con: Poll glands group during indistinct estrus. Exp: Poll glands group during vigorous estrus. Statistical analyses: Wilcoxon rank sum test, α = 0.05.

Analysis of DEPs in the poll gland tissues of male camels during indistinct and vigorous estrus revealed significant findings. As shown in Figure 1, a total of 79,364 precursors, 70,774 peptides, 6901 protein groups, and 7009 proteins were identified (Figure 1A). The distribution analysis of peptide numbers indicates that 32% of proteins are composed of 11 or more peptides, while 11% of proteins contain only 1 peptide (Figure 1B). A total of 1703 DEPs were screened in the experimental group samples compared with the control group, including 709 upregulated proteins and 994 downregulated proteins (Table S2 and Figure 1C). GO annotation revealed that these DEPs are involved in numerous terms and pathways (Figure 1D). The DEPs are predominantly enriched in biological processes related to the inflammatory/immune response and metabolic process, particularly the amino acid and small molecule metabolic process (Table S3 and Figure 1E). Additionally, these DEPs are mainly associated with metabolic pathways and the biosynthesis of amino acids (Table S4 and Figure 1F). The results suggested that the poll glands of male Bactrian Camels exhibit vigorous metabolism during estrus and may contain H_2_S related metabolism.

### 2.2. Identification of Candidate DEPs Related to H_2_S Production

**Figure 2:**
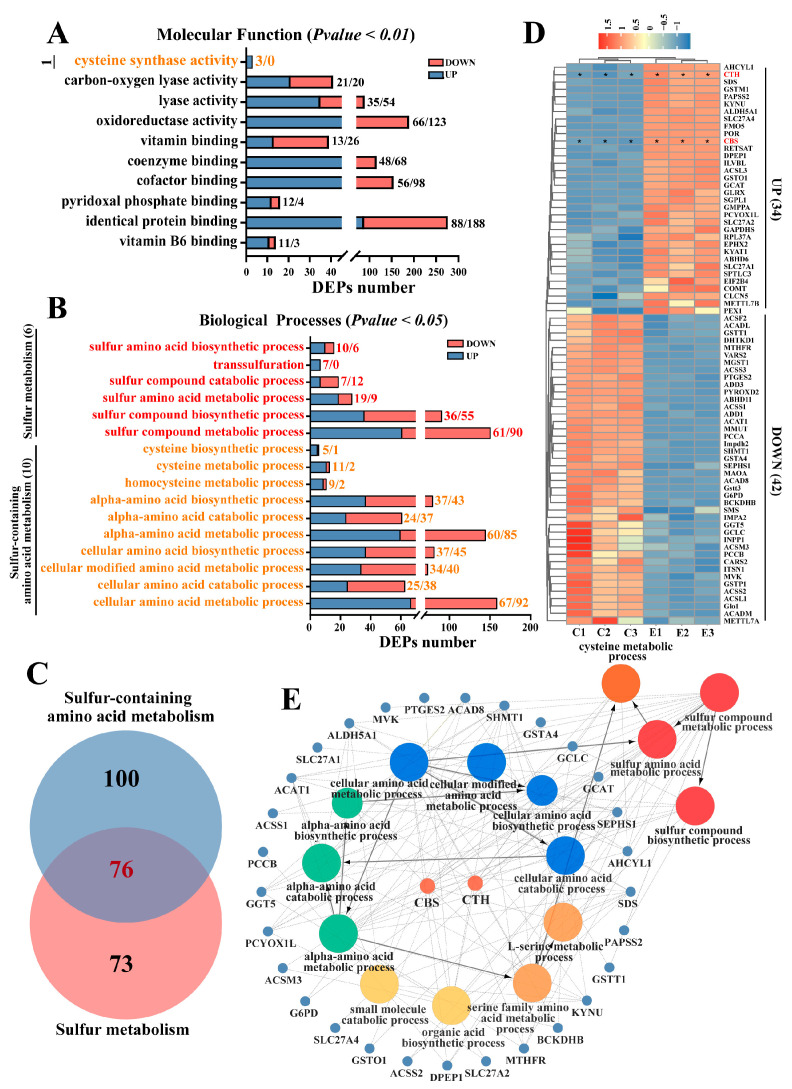
Identification of candidate DEPs related to H_2_S production. Red font indicates GO terms related to sulfur metabolism, while orange font indicates GO terms associated with sulfur-containing amino acidsz. (**A**,**B**). Screen of molecular function term and biological processes terms associated with sulfur and sulfur-containing amino acid metabolism under GO annotation. (**C**) Venn diagram shows differential genes in sulfur metabolism and sulfur amino acid metabolism, respectively. (**D**) Cluster analysis and heat map of these 76 differentially expressed proteins. Red font highlights key enzymes involved in H_2_S synthesis, including CTH and CBS. (**E**) Analysis of 34 upregulated genes by constructing a protein–protein interaction network (PPI). Orange represents sulfur-containing amino acid metabolism processes, red represents sulfur metabolism processes, blue represents cellular amino acid metabolism processes, green represents α- amino acid metabolism processes, and gold and light orange represent small molecule metabolism and serine metabolism processes, respectively. Con: Poll glands group during indistinct estrus. Exp: Poll glands group during vigorous estrus. Statistical analyses: Wilcoxon rank sum test, α = 0.05. Data are presented as median (rank). * Represents *p* < 0.05.

To investigate H_2_S production metabolism in poll gland tissues during estrus, this study further screened candidate differentially expressed genes and pathways related to sulfur and sulfur-containing amino acid metabolism. As shown in Figure 2 and Table S5, one term in molecular function and sixteen terms in biological processes were identified under GO annotation to be associated with sulfur and sulfur-containing amino acid metabolism (Figure 2A,B). A total of 76 DEPs were identified as being associated with both sulfur metabolism and sulfur-containing amino acid metabolism (Figure 2C). Cluster analysis and heat map of these 76 DEPs revealed that 34 proteins were upregulated and 42 proteins were downregulated (Figure 2D and Table S6). Notably, the expression levels of key enzymes CTH and CBS involved in H_2_S biosynthesis in the poll gland tissues of the Exp group were significantly increased compared to the Con group (Figure 2D), suggesting that the H_2_S synthesis pathway in the poll gland tissues of male Bactrian Camels is activated during estrus. Analysis of 34 upregulated proteins through a protein–protein interaction network (PPI) revealed that CTH and CBS play central roles in amino acid metabolism, particularly in sulfur and sulfur-containing amino acid metabolism (Figure 2E). These results indicate that during estrus, the poll gland tissues of Bactrian Camels may promote H_2_S synthesis by activating proteins related to sulfur and sulfur-containing amino acid metabolism, especially the expression of CTH and CBS.

### 2.3. Identification of Candidate DEPs Related to H_2_S Synthesis from the KEGG Pathway

**Figure 3:**
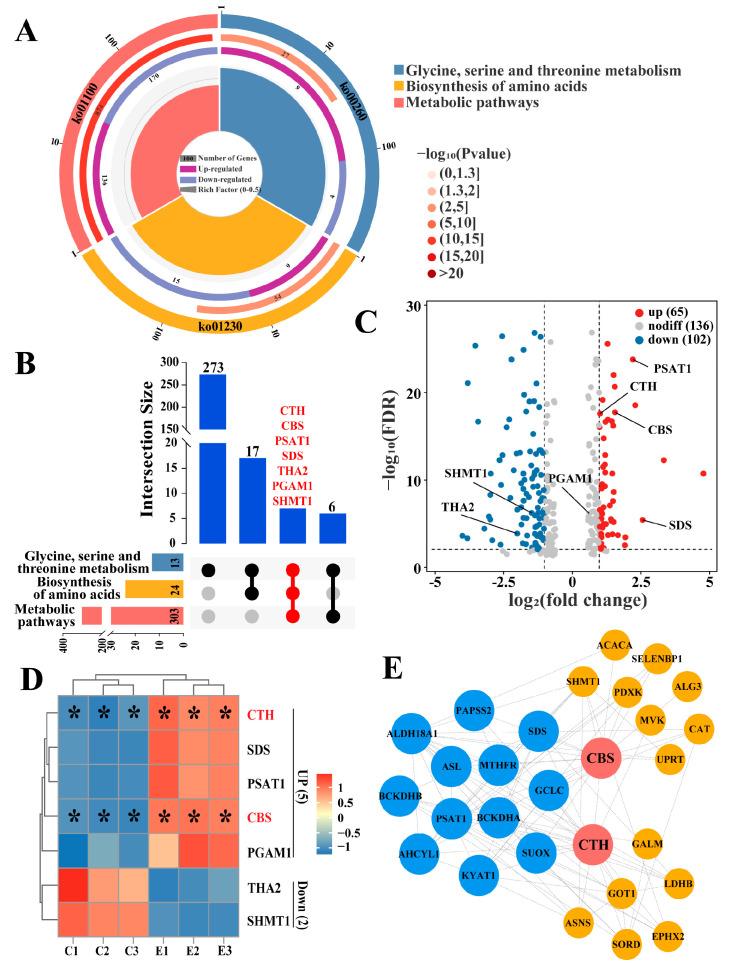
Screening the metabolic pathways involved in CTH and CBS based on KEGG pathway. (**A**) Candidate pathways for CTH and CBS; both involvements were selected from 45 significantly different pathways from KEGG annotation. (**B**) Seven genes were screened to be significantly regulated in all three pathways. (**C**) Volcano plot shows the positions of the 7 genes significantly regulated. Vertical dashed lines are drawn at log_2_(fold change) = −2 and 2, representing the thresholds for significantly down-regulated and up-regulated genes, respectively; The horizontal dashed line is positioned at −log_10_(FDR) = 1.3, indicating the significance threshold of FDR = 0.05. (**D**) Heatmap shows the clustering of the 7 significantly regulated genes. Red font highlights key enzymes involved in H_2_S synthesis, including CTH and CBS. (**E**) The PPI network of DEPs’ interaction with CTH and CBS in three pathways. Yellow represents proteins that interact with CBS or CTH, respectively. Blue represents 12 proteins that interact with both CTH and CBS. Con: Poll glands group during indistinct estrus. Exp: Poll glands group during vigorous estrus. Data are presented as median (rank). * Represents *p* < 0.05.

To explore the roles of CTH and CBS in signaling pathways and regulatory networks, three pathways including glycine, serine and threonine metabolism, biosynthesis of amino acids and metabolic pathways were screened from KEGG analyses (Figure 3A and Table S7). Seven genes, including CTH, CBS, phosphoserine aminotransferase isoform X1(PSAT1), L-serine dehydratase (SDS), probable low-specificity L-threonine aldolase 2 (THA2), phosphoglycerate mutase 1 (PGAM1), and serine hydroxy methyltransferase and cytosolic isoform X1 (SHMT1), were significantly regulated in all three pathways (Figure 3B). THA2 and SHMT1 were downregulated, while CTH, CBS, PSAT1, SDS, and PGAM1 were upregulated (Figure 3C,D). PPI construction revealed that 12 proteins, including SDS and PSAT1, interact with both CTH and CBS. This suggests that CTH and CBS play significant roles in amino acid metabolism and that these interacting proteins may serve as potential factors affecting CTH/CBS-mediated H_2_S synthesis (Figure 3E).

### 2.4. Joint Screening of Protein Factors and Signaling Pathways Related to H_2_S Synthesis

**Figure 4:**
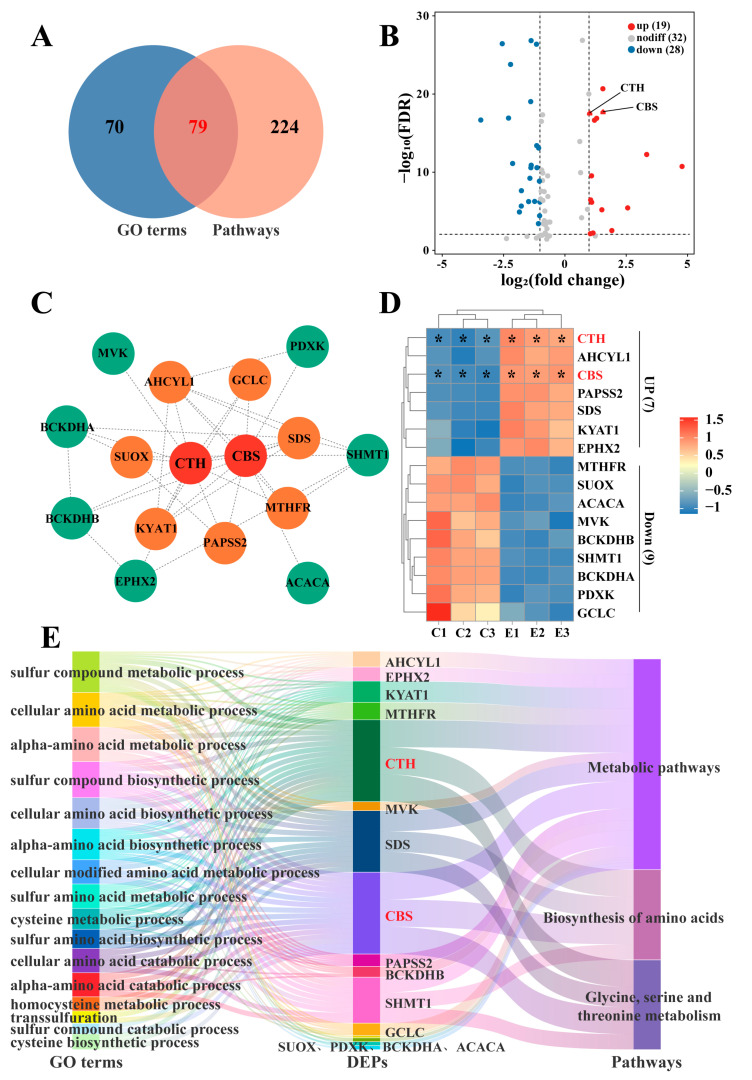
Identification of factors and pathways involved in sulfur metabolism and sulfur-containing amino acid metabolism. (**A**) Venn diagram of DEPs selected from GO terms and KEGG pathways associated with sulfur metabolism and sulfur-containing amino acid metabolism. (**B**) Volcano plot shows the distribution of 79 gene expression levels screened from (**A**). (**C**) Protein–protein interaction (PPI) network construction for the 79 proteins identified in panel (**A**). Orange nodes represent proteins interacting with both CTH and CBS, while green nodes represent proteins interacting with either CTH or CBS. Red nodes denote the key enzymes for H_2_S synthesis, CTH and CBS. (**D**) Heatmap showing the clustering of 16 significantly differentially expressed proteins from 79 proteins. (**E**) Sankey diagram illustrating the relationship between DEPs and three pathways and 16 biological processes related to sulfur metabolism and sulfur-containing amino acid metabolism. Red font highlights key enzymes involved in H_2_S synthesis, including CTH and CBS. Statistical analyses: Wilcoxon rank sum test, α = 0.05. Data are presented as median (rank). * Represents *p* < 0.05.

To further screen for proteins and signaling pathways related to H_2_S synthesis, potential regulatory proteins were screened from 149 DEPs in 16 GO terms and 303 DEPs in three significant pathways. Venn diagrams show that 79 DEPs are potentially related to H_2_S synthesis (Figure 4A). Among them, 19 genes were upregulated and 28 genes were downregulated in the poll glands of male Bactrian Camels during estrus (Figure 4B). PPI analysis of the 79 proteins revealed that SDS, glutamate-cysteine ligase catalytic subunit (GCLC), S-adenosylhomocysteine hydrolase-like protein 1 (AHCYL1), S-adenosylhomocysteine hydrolase-like protein 1 (SUOX), kynurenine--oxoglutarate transaminase 1 isoform X1 (KYAT1), bifunctional 3′-phosphoadenosine 5′-phosphosulfate synthase 2 (PAPSS2), and methylenetetrahydrofolate reductase isoform X1 (MTHFR) interact with both CTH and CBS. Additionally, mevalonate kinase (MVK), 2-oxoisovalerate dehydrogenase subunit alpha (BCKDHA), 2-oxoisovalerate dehydrogenase subunit beta (BCKDHB), and bifunctional epoxide hydrolase 2 (EPHX2) interact with CTH, while pyridoxal kinase isoform X1 (PDXK), SHMT1, and acetyl-CoA carboxylase 1 isoform X3 (ACACA) interact with CBS (Figure 4C and Table S8). These interactions suggest that these proteins may serve as potential factors influencing CTH/CBS-mediated H_2_S synthesis. The heatmap indicated that the expression levels of most of these 16 proteins are inversely correlated with CTH and CBS (Figure 4D), suggesting that most of these genes negatively regulate CTH and CBS expression and H_2_S synthesis. The Sankey diagram constructs the relationship between DEPs and three pathways and 16 biological processes related to sulfur metabolism and sulfur-containing amino acid metabolism (Figure 4E), confirming the important role of these genes in sulfur metabolism and sulfur-containing amino acid metabolism. These results further indicate that CTH and CBS play essential roles in amino acid metabolism and that numerous regulatory factors are involved in H_2_S synthesis.

### 2.5. Identification of Differentially Expressed Metabolites (DEMs) Involved in H_2_S Synthesis Based on GC-MS

**Figure 5:**
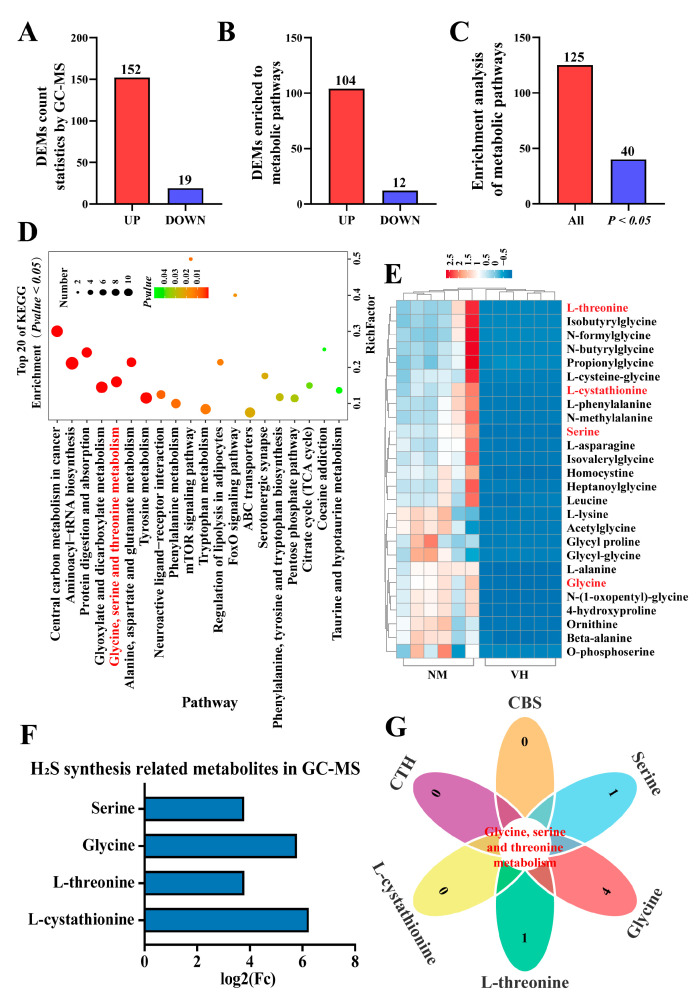
Identification of differentially expressed metabolites (DEMs) involved in H_2_S synthesis based on GC-MS analysis. (**A**) The number of DEMs identified in neck hair of male Bactrian Camels during estrus compared to ventral hair using non-targeted metabolomics analysis via the GC-MS method. (**B**) The number of DEMs enriched in metabolic pathways using KEGG annotation. (**C**) The number of pathways to which DEMs were mapped and enriched (*p* < 0.05) using KEGG annotation. (**D**) The top 20 enriched pathways in (**C**). (**E**) Heatmap showing the differential content of 26 amino acids using GC-MS analysis. NM: neck hair; VH: ventral hair. Red font indicates important DEMs in the glycine, serine, and threonine metabolic pathways, which are significant in relation to H_2_S synthesis. (**F**) The changes in cystathionine, glycine, serine, and threonine levels, related to H_2_S synthesis, in neck hair compared to ventral hair. (**G**) Venn diagram showing that CBS, CTH and cystathionine, glycine, serine, and threonine share the same metabolic pathway. Data are presented as median (rank).

To further explore potential H_2_S metabolism in the poll gland tissues of male Bactrian Camels during estrus, this study collected the ventral hair and neck hair of male Bactrian Camels during estrus for non-targeted metabolomics analysis using the GC-MS method. As shown in Figure 5, 171 DEMs were identified in the neck hair of male Bactrian Camels during estrus, of which 152 were upregulated and 19 were downregulated (Figure 5A). A total of 116 DEMs were enriched in the metabolic pathways, including 104 upregulated DEMs and 12 downregulated DEMs (Figure 5B). These DEMs were mapped to 125 signaling pathways, with 40 pathways highly enriched (*p* < 0.05, Figure 5C). KEGG annotation indicated that glycine, serine, and threonine metabolism are important differential pathways (Table S9 and Figure 5D). Cluster analysis and heat map showed that the levels of various amino acids, including cystathionine, glycine, serine, and threonine, were significantly higher in the neck hair group compared to the ventral hair group (Figure 5E,F and Table S10). Together with the result that CTH and CBS are essential in glycine, serine, and threonine metabolism and H_2_S biosynthesis (Figure 5G), these results indicated that amino acid metabolism, especially related to H_2_S biosynthesis, is very active in the poll gland tissues of Bactrian Camels during estrus, and CTH and CBS may regulate amino acid metabolism and H_2_S synthesis through glycine, serine, and threonine metabolic pathways.

### 2.6. Expression Pattern and Distribution of CTH and CBS in Poll Gland Tissues

**Figure 6:**
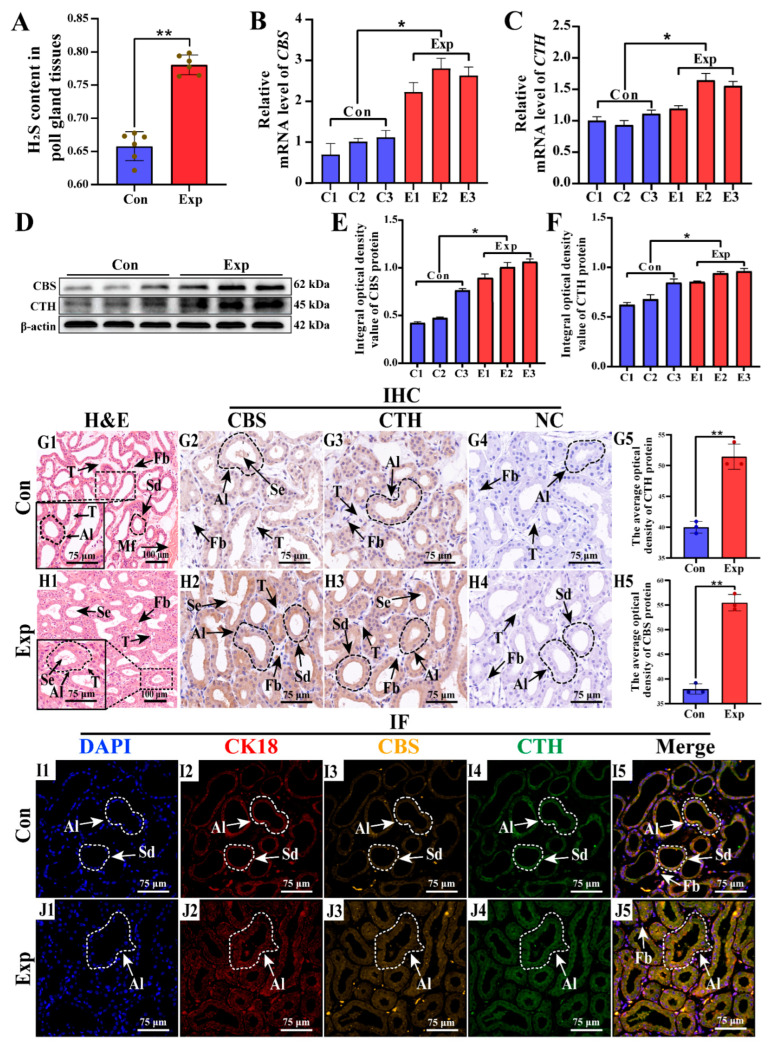
Concentration of H_2_S and the expression and distribution of CTH and CBS in poll gland tissues. (**A**) Detection of H_2_S content in poll gland tissues. (**B**,**C**) The mRNA levels of *CBS* and *CTH* monitored by qRT-PCR assays. (**D**–**F**) The protein expression levels of CTH and CBS monitored by Western blot assay and the optical density of bands. (**G1**,**H1**) Histological staining of poll glands using the H&E method. (**G2**–**G4**,**H2**–**H4**) The IHC staining of poll glands against CBS and CTH, respectively. (**G5**,**H5**) The gray values of positive expression of CTH and CBS proteins with IHC sections were scanned and quantified. (**I1**,**J1**) DAPI-labeled nuclei of cells from various cell types in cervical gland tissues of groups Con and Exp. (**I2**,**J2**) Epithelial cell-specific marker CK-18 observed in the cytoplasm of poll glands epithelial cells. (**I3**,**I4**,**J3**,**J4**) Cellular localization of CTH and CBS. (**I5**,**J5**) Co-localization analysis showing CK-18, CTH, and CBS in the cytoplasm of poll glands epithelial cells. Con: Poll glands group during indistinct estrus. Exp: Poll glands group during vigorous estrus. NC: negative control. AI: acinar. Sd: secretory duct. Se: secretions. Mf: muscle fibers. Fb: Fibroblasts. Data are presented as means  ±  SEM. * represents *p* < 0.05 and ** represents *p* < 0.01.

To verify whether H_2_S is synthesized in the poll gland tissues of male Bactrian Camels during estrus and to examine the expression pattern and distribution of CTH and CBS, the H_2_S content, as well as the protein localization and gene expression levels of CTH and CBS, were assessed by H_2_S level assay, qRT-PCR, Western blotting, and IHC and IF assays, respectively. As shown in Figure 6, H_2_S levels were found to be significantly elevated in poll gland tissues by H_2_S assay (Figure 6A and Table S11). The mRNA and protein expression levels of CBS and CTH in the poll gland tissues of male Bactrian Camels during vigorous estrus were significantly elevated, as determined by qRT-PCR and Western blot analysis, respectively (Figure 6B–F and Table S12). These results are consistent with the DIA proteomics data, indicating that the expressions of CTH and CBS were activated in the poll gland tissues of male Bactrian Camels during vigorous estrus. Additionally, H&E staining showed a dense distribution of small tubules, including acinar and secretory ducts, in poll gland tissues. During vigorous estrus, the acinar cells in the poll gland tissues significantly increased in size and quantity, with an increase in secretions in the tubules and a decrease in connective tissue (Figure 6(G1,H1)). The IHC staining showed that CTH and CBS were mainly distributed in the cytoplasm of acinar cells, and the staining of CTH and CBS in the vigorous estrus poll glands was significantly deeper than that in the control tissue (Figure 6(G2–G5, H2–H5)). Consistently, IF staining also showed higher expression of CTH and CBS in the poll glands during vigorous estrus (Figure 6(I1–I4, J1–J4)). Additionally, CTH and CBS showed co-localization in acinar cells (Figure 6(I5, J5)). These results suggested that the expression of CTH and CBS is activated in the poll glands of male Bactrian Camels during estrus, which may result in more active amino acid metabolism and an enhanced ability to synthesize H_2_S.

## 3. Discussion

Bactrian Camels are seasonal estrus animals, and the development and secretion of the poll glands in male camels are closely related to their sexual activity [5]. However, current research on the function of the poll gland tissues in Bactrian Camels is very limited. The gaseous signaling molecule H_2_S is believed to play an important role in multiple physiological processes, including vasodilation and nerve conduction [12]. Despite this, the function and regulatory mechanism of H_2_S in the poll glands of male Bactrian Camels remain unclear. To elucidate the synthesis and mechanism of H_2_S in the poll gland tissues of Bactrian Camels, DIA proteomics analysis was performed to identify and screen differentially expressed proteins. DIA proteomics is a comprehensive, reproducible, and precise method that has been used to study the mechanisms of various physiological processes [17,18]. Increasing evidence has suggested that, beyond their roles as components of proteins and peptides, certain amino acids such as arginine, cysteine, glutamine, leucine, proline, and tryptophan are crucial regulators of key metabolic pathways essential for maintaining growth, reproduction, and immunity in organisms [19]. Research indicates that H_2_S can be produced through various mechanisms involving L-homocysteine and cysteine through the methionine sulfur conversion pathway, and the metabolism of sulfur-containing amino acids in the body determines the concentration of H_2_S products [20].

This study identified protein factors related to endogenous H_2_S synthesis in the poll gland tissues of Bactrian Camels during estrus using DIA proteomics technology, It was found that the expression of two key enzymes, CTH and CBS, was significantly increased. Endogenous H_2_S plays a crucial role in hormone responsiveness and estrus regulation in mammals. Estradiol, a hormone that induces estrus, can rapidly stimulate the release of H_2_S from endothelial cells [21,22]. H_2_S can significantly alleviate spermatogenesis disorders caused by inflammation and oxidative stress, restore testosterone synthesis in vitro and in vivo, and maintain testicular function [23]. Therefore, we hypothesize that H_2_S synthesis in the poll glands of male Bactrian Camels may have an estrus-inducing effect. Based on DIA proteomics data, this study identified 76 DEPs related to sulfur metabolism and sulfur-containing amino acid metabolism via GO annotation, including CTH and CBS. Notably, the glycine, serine, and threonine metabolic pathway, which involves CTH and CBS, is a key metabolic pathway for synthesizing H_2_S [20,24]. Non-targeted metabolomics data also indicated that the levels of glycine, threonine, serine, and cystathionine significantly increase in poll gland tissues, suggesting that the glycine, serine, and threonine metabolic pathway is very active in poll glands. Expression pattern studies revealed that CTH and CBS were widely distributed in the acinar cells of poll glands, and their expression increased significantly during estrus, which may lead to vigorous H_2_S synthesis metabolism.

**Figure 7:**
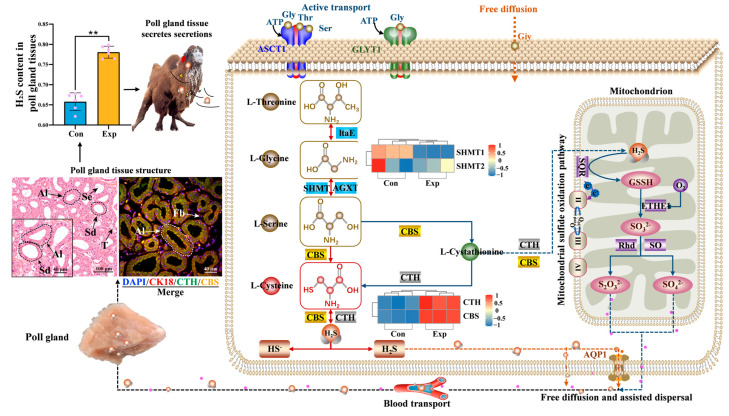
Potential molecular mechanism of H_2_S synthesis mediated by CTH and CBS in the poll glands of male Bactrian Camels. ** represents *p* < 0.01.

In summary, as shown in Figure 7, after exogenous amino acids are absorbed and transported to tissues via the bloodstream, glycine, threonine, and serine are actively transported into cells through the SLC1 amino acid transporter family member ASCT1 (SLC1A4) on the cell membrane [25,26,27]. Under the action of L-threonine aldolase (ltaE), serine hydroxy-methyltransferase (SHMT), serine pyruvate transaminase (AGXT), and other enzymes, including glycine and threonine, are converted to L-serine [28,29], which is further converted to L-cysteine for H_2_S synthesis via the action of CBS. Through the joint action of CBS and CTH, L-cysteine is ultimately metabolized to produce H_2_S, which exerts physiological effects [28,30]. H_2_S exists in dynamic equilibrium within cells in both H_2_S and HS forms [10]. Excess H_2_S is cleared by entering the mitochondria as intermediate cystathionine under the action of CBS and CTH and converting into sulfates (SO_4_^2−^) and thiosulfate (S2O_3_^2−^), mainly catalyzed by sulfoquinone oxidoreductase (SQR), peroxydisulfide dioxygenase (ETHE1), and sulfite oxidase (SO) or rhodanese (Rhd) [13,14,31]. The electrons released in the SQR catalyzed reaction are captured by ubiquinone (Coenzyme Q, CoQ) and transferred to the electron transport chain in complex III, indicating that the oxidation and elimination of H_2_S can promote adenosine triphosphate (ATP) synthesis. Some H_2_S diffuses into the extracellular space with the assistance of the transmembrane receptor aquaporin 1 (AQP1) [15,16]. One thing to keep in mind is that the existence of SLC1A4, AQP1, ltaE, and AGXT in camels is inferred solely from homology, while the existence of ETHE1 and SO is only predicted. There is no experimental evidence for the existence of GLYT1 and SQR in camels. Consequently, the hypothesis presented in Figure 7 is speculative, as only two proteins, CTH and CBS, have been confirmed to be expressed. Combined with the observation that the H_2_S concentration in the poll glands of Bactrian Camels during estrus significantly increases, these results suggest that the poll glands of male Bactrian Camels can release H_2_S, thereby inducing estrus in females. However, further experimental validation is necessary to confirm these pathways and protein expressions in camels.

## 4. Materials and Methods

### 4.1. Sample Collection and Processing

All animal samples were collected strictly in accordance with the Animal Ethics Regulations (GSU-LC-2020-39) approved by the Animal Protection Committee of Gansu Agricultural University. Due to the difficulty of sample collection caused by the atrophy and collapse of the poll gland tissues during the non-estrus period, the experimental samples in this study were collected from the poll gland tissues of a free-ranging male Bactrian Camel in January estrus from a farmer in Zhangye City, Gansu Province, China. As previously mentioned, the samples were evaluated based on the camel’s estrus state (foaming at the mouth, beeping, grinding teeth, “water whipping”, running, fighting for mate, itching, loss of appetite, etc.) and the amount of poll gland secretion [5]. The vigorous estrus (Exp/E) group (*n* = 3) consisted of male camels with strong poll gland secretion, aged 8 years and weighing about 480 kg, while the indistinct estrus (Con/C) group (*n* = 3) consisted of male camels with no obvious secretion. After slaughtering camels, sterile poll glands tissue samples were collected with fixation with 4% paraformaldehyde for histochemistry analysis and freezing at −80 °C for proteomic analysis, respectively. Ventral hair (VH) and neck mane (NM) samples were collected from each male camel in the study group (*n* = 6) and subjected to untargeted metabolomic analysis.

### 4.2. DIA Proteomics

Sample pre-treatment included protein extraction, denaturation, reductive alkylation, enzymatic lysis, and peptide desalting [32,33]. Using the PreOmics iST Sample Pretreatment Kit, samples were ground in liquid nitrogen, lysate was added, and the mixture was heated at 95 °C for 10 min. After cooling, trypsin digestion buffer was added and samples were incubated at 37 °C for 2 h with oscillation. After termination of the enzymatic reaction, peptides were desalted using the iST cartridge, eluted, and vacuum-dried for storage. To create a spectral database, the peptide mixture was first dissolved in ammonium formate buffer and separated by high pH reversed-phase separation using an Ultimate 3000 system and an XBridge C18 column, with a linear gradient from 5% B to 45% B in 40 min. Six segments were collected and dried for use. The re-solubilized peptides were then analyzed by low pH nano-HPLC-MS/MS using an LC-MS/MS system equipped with an on-line nanojet ion source and an Orbitrap Lumos mass spectrometer (Thermo Fisher Scientific, Waltham, MA, USA) paired with an EASY-nLC 1200 system, operating in data-dependent acquisition mode, with the appropriate mass spectrometry parameters set for MS and MS/MS data acquisition. Finally, the raw data were merged and analyzed using Spectronaut X software (version 18, Biognosys) to search Uniprot or provided databases, assessing sample contamination and setting the appropriate search library parameters. Suspensions were made from each sample, and after the addition of the iRT peptides, they were separated using nano-LC and analyzed by tandem mass spectrometry for DIA data acquisition. The mass spectrometry parameter settings included scan range, resolution, AGC target value, maximum injection time, and collision energy. Variable window acquisition was used, with 60 windows set up, each overlapping by 1 *m*/*z*. The *Q*-value (FDR) threshold was set at 1% for both precursor and protein levels. Precursors that passed these filters were used for quantification. Major group quantities were calculated using the average of the top three peptides that passed the 1% *Q*-value threshold. DEPs were identified by applying Student’s *t*-test, with criteria set at *Q* < 0.05 and an absolute log2 ratio > 0.58. The DIA proteomics data have been deposited in ProteomeXchange under accession number PXD047457.

### 4.3. Metabolomics of GC-MS

The 30 mg NM and VH tissues samples were accurately weighed and placed into 1.5 mL centrifuge tubes, to which 20 μL of internal standard (L-2-chlorophenylalanine, 0.06 mg/mL in methanol) and 600 μL of methanol–water (4:1, *v*/*v*) were added. The samples were held at −80 °C for 2 min and then ground at 60 Hz for 2 min. Subsequently, 120 μL of chloroform was added, followed by vortexing for 2 min and ultrasonic extraction in an ice-water bath for 10 min. The samples were then left to stand at −20 °C for 30 min. Centrifugation was performed at 13,000 rpm for 10 min at 4 °C, and 150 μL of the supernatant was transferred to a glass derivatization vial and evaporated to dryness using a centrifugal concentrator. Then, 80 μL of methoxyamine hydrochloride in pyridine (15 mg/mL) was added, vortexed for 2 min, and incubated at 37 °C for 90 min to allow oximation. After this reaction, 50 μL of BSTFA (with 1% TMCS) and 20 μL of n-hexane were added, along with 10 μL of an internal standard mixture (C8–C24); the mixture was vortexed for 2 min, and then reacted at 70 °C for 60 min. The samples were then allowed to stand at room temperature for 30 min before GC-MS analysis. Quality control (QC) samples were prepared by pooling equal volumes of extracts from all samples. Metabolomic analysis was performed using an Agilent 7890B-5977A gas chromatography–mass spectrometry (GC-MS) system (Agilent J&W Scientific, Folsom, CA, USA). The conditions for GC-MS analysis, including the temperature program and mass spectrometry settings, were as previously described [5]. The multivariate statistical analysis first used unsupervised Principal Component Analysis (PCA) to observe the overall distribution among the samples and the stability of the whole analysis process, and then supervised Partial Least Squares Analysis (PLS-DA) and Orthogonal Partial Least Squares Analysis (OPLS-DA) were used to differentiate the overall differences in metabolic profiles among the groups, to find the differential metabolites among the groups, and to ensure the quality of the model by 7 cross validation and 200 alignment tests to ensure the quality of the model. Metabolites showing a Variable Importance in Projection (VIP) value > 1 and a *p*-value < 0.05 were selected as significantly different [5]. Pathway enrichment analysis was conducted using KEGG, with pathways showing a *p*-value ≤ 0.05 considered significantly different.

### 4.4. Bioinformatics Analysis

DIA proteomics was used to identify DEPs associated with endogenous H_2_S synthesis. GC-MS was used to identify DEMs associated with endogenous H_2_S synthesis. Gene Ontology (GO) annotation and Kyoto Encyclopedia of Genes and Genomes (KEGG) enrichment were performed to analyze differentially expressed genes with the screening criteria of *p* < 0.05. Heatmaps, Venn diagrams, and other visualizations related to DEPs and DEMs were created using the OmicShare online platform (https://www.omicshare.com/tools), accessed on 7 April 2023. Candidate protein interaction networks were generated using STRING v.10.0 (https://cn.string-db.org (accessed on 7 April 2023)) and Cytoscape (version 2.8.1). Signal transduction maps for H_2_S synthesis and metabolism were conducted using Adobe Illustrator 2020 (San Jose, CA, USA).

### 4.5. Endogenous H_2_S Detection

Endogenous H_2_S concentration in poll gland tissues was measured using the Micro H_2_S Content Assay Kit (Solarbio, Beijing, China) according to the manufacturer’s instructions. The experiment was conducted as previously described [34]. All H_2_S detection was performed using a microplate reader (ReadMax 1900, Shanghai, China) at a wavelength of 665 nm. All experiments were repeated at least three times. 

### 4.6. Histochemistry Staining

Fixed tissue was embedded in paraffin and sliced into 5 µm thick sections using a slicer (Leica, Wetzlar, Germany). Hematoxylin and eosin (H&E) and immunohistochemical (IHC) staining were conducted as previously described [35,36]. Rabbit anti-CBS primary antibody (1:150, Abcam, Cambridge, UK) and rabbit Anti-CTH antibody (1:100, Bioss, Beijing, China) were used. Signal images were captured using a panoramic desk scanner system (3D HISTECH, Budapest, Hungary). All experiments were conducted three times.

### 4.7. Immunofluorescence (IF) Staining 

The IF staining was performed as described previously [35]. Rabbit anti-CBS primary antibody (1:200, Abcam, Cambridge, UK), rabbit Anti-CTH antibody (1:200, Bioss, Beijing, China), and anti-cytokeratin 18 (CK18) primary antibody (1:300, Bioss, Beijing, China) were used. Cell nuclei were labeled with 40,6-diamidino-2-phenylindole (DAPI). Fluorescence signals were captured using a fluorescence microscope (Olympus, Tokyo, Japan). All experiments were conducted three times.

### 4.8. RNA Extraction, cDNA Synthesis and qRT-PCR Detection

The total RNA extraction, cDNA synthesis, and qRT-PCR detection were performed as described earlier [36,37]. The qRT PCR primers (Table S1) were designed using Premier 5.0 software and synthesized by Qinke Biotech Co., Ltd. (Yangling, Shaanxi, China). The expression levels of glyceraldehyde-3-phosphate dehydrogenase (*GAPDH*) were used as the internal reference control. The relative expression level of candidate genes was evaluated using the 2^−ΔΔCT^ method. All tests were repeated at least three times.

### 4.9. Western Blot Assay

Western blot assay were used to detect the expression levels of candidate proteins in tissue samples as described earlier [36,37]. The primary antibodies were incubated at different dilution factors at 4 °C overnight. Rabbit anti-CBS primary antibody (1:3000, Abcam, Cambridge, UK), rabbit Anti-CTH antibody (1:1000, Bioss, Beijing, China). Anti-β-actin primary antibody (1:4500, Bioss, Beijing, China). The bands density values were quantified by Image-Pro Plus 6.0 (Media Cybernetics, Rockville, MD, USA). All tests were conducted at least three times.

### 4.10. Statistical Analysis

Unless otherwise specified, all data are presented as mean ± SEM. Statistical analysis of the data was performed by SPSS (v22.0, Chicago, IL, USA) using Student’s *t*-test (between two groups) or one way ANOVA (within multiple groups). Data graphs were generated using GraphPad Prism (v9.0, San Diego, CA, USA). *p* values < 0.05 were considered significant.

## 5. Conclusions

In this study, we utilized DIA proteomics analysis to determine that the key enzymes for endogenous H_2_S synthesis, CTH and CBS, were significantly upregulated, indicating the potential synthesis of endogenous H_2_S in the poll gland tissue of male Bactrian Camels. Comprehensive screening of proteins and LC-MS differential metabolites related to sulfur metabolism and amino acid pathways highlights the importance of glycine, serine, and threonine metabolic pathways in H_2_S synthesis. Additionally, CTH and CBS are widely distributed in poll glands’ acinar cells, especially during estrus, indicating their association with vigorous H_2_S synthesis metabolism. Finally, this study proposes a mechanism for the endogenous synthesis of H_2_S and provides a theoretical basis for exploring the physiological functions of H_2_S in the poll gland tissues of male Bactrian Camels.

## Data Availability

The data that support the findings of this study are available from the corresponding author upon reasonable request.

## Supplementary Materials

The following supporting information can be downloaded at: https://www.mdpi.com/article/10.3390/ijms25147700/s1.
